# Mitochondrial DNA: Consensuses and Controversies

**DOI:** 10.3390/dna2020010

**Published:** 2022-06-10

**Authors:** Inna Shokolenko, Mikhail Alexeyev

**Affiliations:** 1 Department of Biomedical Sciences, Pat Capps Covey College of Allied Health Professions, University of South Alabama, Mobile, AL 36688, USA; 2 Department of Physiology and Cell Biology, University of South Alabama, Mobile, AL 36688, USA

**Keywords:** mtDNA, mtDNA transcription, mtDNA replication, mtDNA repair, mitochondrial theory of aging, extramitochondrial mtDNA

## Abstract

In the course of its short history, mitochondrial DNA (mtDNA) has made a long journey from obscurity to the forefront of research on major biological processes. mtDNA alterations have been found in all major disease groups, and their significance remains the subject of intense research. Despite remarkable progress, our understanding of the major aspects of mtDNA biology, such as its replication, damage, repair, transcription, maintenance, etc., is frustratingly limited. The path to better understanding mtDNA and its role in cells, however, remains torturous and not without errors, which sometimes leave a long trail of controversy behind them. This review aims to provide a brief summary of our current knowledge of mtDNA and highlight some of the controversies that require attention from the mitochondrial research community.

## 1. Introduction

The metazoan genome is sequestered in two spatially distinct compartments: the nucleus and the mitochondria. The nuclear genome encodes the vast majority of genetic information and is represented by two chromosomes of each type inherited biparentally (one from each parent). In contrast, the metazoan mitochondrial genome is typically a much smaller (~14,000–18,000 bp), maternally inherited circular molecule, present in multiple copies, from less than a dozen per mature human sperm [1] to more than 100,000 in oocytes [2].

Discovered almost 60 years ago by Nass and Nass [3], mitochondrial DNA (mtDNA) for a long time was considered vestigial until 1988, when several groups established a link between mtDNA mutations and incurable, devastating, and often lethal human diseases, for which we still do not have effective treatments [4–6]. These discoveries brought about an era of mitochondrial biology in which mtDNA plays a central role as it contributes to all mitochondrial functions, either directly or indirectly.

Over the years, mtDNA alterations have been implicated in the pathogenesis of virtually all organ systems: respiratory system [7,8], digestive and excretory system [9,10], circulatory system [11,12], urinary system [13], integumentary system [14,15], skeletal system [16,17], muscular system [18], endocrine system [18], lymphatic system [19,20], nervous system [21,22], and reproductive system [23–25]. Due to this ubiquitous involvement in biological processes, mtDNA elicits widespread interest. As a result of this interest, most of today’s knowledge on mtDNA has been derived from human and related mammalian and vertebrate species. Unless otherwise indicated, this review will be limited to these species.

## 2. mtDNA Organization

**Figure 1.**
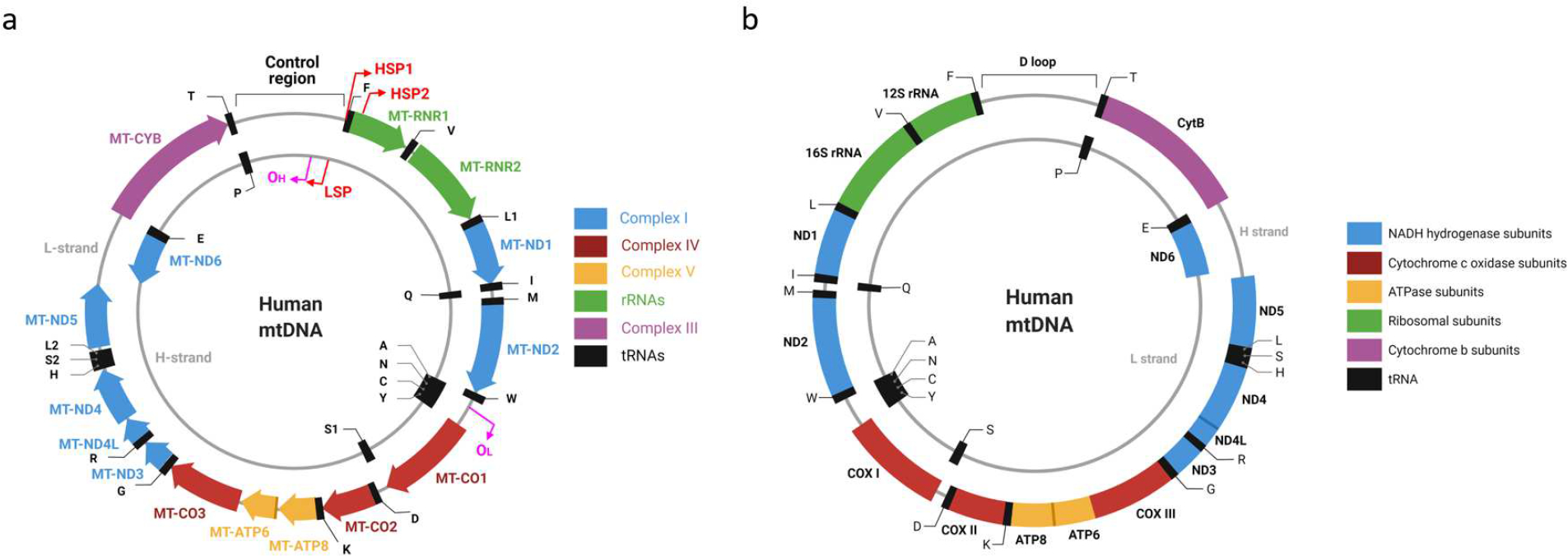
Alternative maps of human mitochondrial DNA (mtDNA). (**a**), a conventional map with clockwise annotation according to the revised Cambridge Reference Sequence (rCRS, GenBank NC_012920). Magenta bent arrows designate origins and direction of new H-strand (OH) and L-strand (OL) synthesis during replication, respectively. Red bent arrows designate mitochondrial promoters that direct transcription of genes encoded in either L-strand (HSP1 and HSP2) or H-strand (LSP), respectively. To avoid crowding, transfer RNAs (tRNAs) are designated by black letters using single-letter codes for corresponding amino acids. (**b**), an alternative map. Note the opposite gene order, misattribution of genes to either H- or L-strands, and HGNC-noncompliant nomenclature of mitochondrial genes. Both maps were generated using BioRender software.

Most commonly, mtDNA is a circular molecule present in cells in a tissue-specific number of copies [26] (Figure 1). The first sequenced mitochondrial genomes were those of humans and mice [27,28], which are of similar length and have identical organization. Each genome encodes 37 genes: 2 rRNA, 22 tRNA, and 13 polypeptides, all of which are components of the mitochondrial oxidative phosphorylation (OXPHOS) system. Seven polypeptides contribute to OXPHOS complex I (CI), one polypeptide (cytochrome B) contributes to complex III (CIII), three polypeptides contribute to complex IV (CIV), and two polypeptides contribute to complex V (CV). Notably, none of the complex II (CII) subunits are encoded in mtDNA; therefore, mtDNA mutations should not affect CII activity. This consideration is used to normalize experimentally determined activities of other OXPHOS complexes, especially when one is studying the primary mitochondrial disease.

Two strands of mtDNA have asymmetric nucleotide composition and, as a result, could be separated in alkaline denaturing CsCl gradients based on their G+T (as opposed to separation based on G+C content observed with dsDNA). This is believed to be because, in alkaline solutions, G and T bases become ionized and can interact with Cs+ ions, thus conferring a higher density to the strand with a higher content of these bases [29]. Therefore, a heavy (high G+T content) and light (low G+T content) strand can be identified in each mtDNA molecule (the H-strand and L-strand, respectively). This distinction between the two mtDNA strands, in conjunction with the asymmetric distribution of genes between strands, is associated with the first mtDNA controversy.

Siv Anderson’s and Clayton’s groups, which sequenced the first mitochondrial genomes [27,28], used different definitions for “coding strand”. Anderson et al. defined the coding strand much like we do today, as the DNA strand whose base sequence is identical to the base sequence of the RNA transcript. They reported that L-strand is the main coding strand in human mtDNA (hmtDNA). It encodes 12 of 13 polypeptides, both ribosomal RNAs (rRNAs), and 14 of 22 transfer RNAs (tRNAs). Bibb et al. called the opposite strand (the template or noncoding strand by today’s convention) coding, and they concluded that the H-strand encodes most genes. Despite being at odds with the contemporary terminology, statements that the H-strand is the main coding strand in hmtDNA can be found even in recent reviews [30]. Interestingly, the L-strand of mtDNA is not the main coding strand in all organisms. A recent review examined 4205 vertebrate mitogenomes and established that the H-strand was the main coding strand in five of them [31].

The confusion about coding mtDNA strands also contributes to the disparate presentation of mtDNA molecules in the literature. Conventionally, circular genomes are annotated clockwise. However, in the mitochondrial literature, it is not uncommon to see mtDNA maps with inverted gene order in addition to the conventional maps that can be automatically generated by software packages from GenBank entries (similar to the one present in Figure 1A). This situation is not helped by the fact that some scientific illustration software makers adopted an outdated counterclockwise depiction of mtDNA (see, e.g., Figure 1B and [32]).

Inside mitochondria, mtDNA is organized into nucleoids named for their resemblance to the irregularly shaped regions within the cell of a prokaryote containing all or most of the genetic material. Apart from mtDNA, these structures contain various proteins that facilitate mtDNA compaction and metabolism [33].

Most commonly, nucleoids are visualized by labeling with various DNA stains, including anti-DNA antibodies, BrdU/anti-BrdU antibodies, and fluorescent intercalators, such as DAPI and Pico Green, etc., followed by fluorescence microscopy. Therefore, the number of nucleoids detected per cell (and thus estimates of the number of mtDNA molecules per nucleoid) depends on the properties of the optical system used, such as optical resolution and signal-to-noise ratio. As a result, reported numbers of mitochondrial genomes per nucleoid greatly vary in the literature. The lowest reported estimate of 1.45 mtDNA molecules per nucleoid, obtained with the help of stimulated emission depletion (STED) microscopy [34], is consistent with the “resting” ratio of one mtDNA molecule per nucleoid and is likely the most accurate. However, it is not possible to exclude the chance that this number is variable and/or depends on the cell type/physiological condition.

Nucleoids are ovoid structures that are diverse in size, with an average diameter of about 100 nm [34,35]. They are associated with the mitochondrial inner membrane and are often wrapped around cristae or cristae-like membrane invaginations [35]. Experimental evidence suggests that there is little, if any, exchange of mtDNA between nucleoids [36]. mtDNA in nucleoids is packed more densely than in *Escherichia coli* nucleoids or human nuclei [37]. mtDNA compaction in nucleoids is driven by mitochondrial transcription factor A (TFAM), a high mobility group (HMG)-box DNA binding protein with functions in mtDNA packaging, replication, and transcription [38]. TFAM’s reported footprint on DNA is 23 bp or 30 bp [39]. Its reported abundance in mitochondria exceeds that of mtDNA by a factor of 1000. This high molar excess of TFAM is sufficient to completely coat mtDNA, assuming that most TFAM is bound to it [34]. This latter assumption is supported experimentally, as it has been reported that TFAM not bound to mtDNA is subject to phosphorylation at Ser55 and Ser56 and degradation by Lon protease [40].

TFAM binds mtDNA specifically at mitochondrial promoters (the L-strand promoter, LSP, and the H-strand promoter 1, HSP1) to facilitate transcription and replication. It also binds the mitochondrial genome non-specifically [41] to induce mtDNA compaction. This compaction depends on TFAM dimerization, which, in turn, is promoted by its N-terminal HMG domain [42]. Whether bound to mtDNA specifically or non-specifically, TFAM imposes on it a sharp bend, dubbed a U-turn. This bending is essential for both transcription and packaging [42].

## 3. mtDNA Nomenclature

Even though mtDNA encodes only 37 genes, their nomenclature remains discordant, controversial, and at times confusing. At least two nomenclatures for mtDNA-encoded genes co-exist in the literature [43,44]. However, the HUGO Gene Nomenclature Committee (HGNC) recommends the one based on the system first implemented by D. Wallace [43,45]. This nomenclature takes historical precedence over its alternative and boils down to a few simple rules. Every mitochondrial gene receives a prefix MT to distinguish it from nuclear genes. This prefix is followed by a hyphen and a gene name. Gene names are *RNR1* and *RNR2* for 12S and 16S mitochondrial rRNAs, respectively, *ND1-ND6* for mtDNA-encoded CI subunits, *CO1-CO3* for CIV subunits, *APT6* and *ATP8* for CV subunits, and *CYB* for the cytochrome B gene. Mitochondrial tRNA gene names consist of the letter T followed by a single-letter amino acid code for the amino acid that acylates this tRNA (e.g., *MT-TV* for mitochondrial valine tRNA). The amino acids leucine and serine acylate two isoacceptor tRNAs, and two corresponding isoacceptor tRNAs are designated *MT-TL1* and *MT-TL2* (for tRNAs recognizing codons UUR and CUN, respectively), and *MT-TS1* and *MT-TS2* (for tRNAs recognizing codons UCN and AGY, respectively). Strikingly, the alternative nomenclature [44] provides the opposite designations to leucine and serine isoacceptor tRNAs (i.e., *L2* or *trnL2* for *MT-TL1* and vice versa). As both nomenclatures have been implemented in software packages, care must be taken when automatically annotating newly sequenced mitochondrial genomes.

Another issue lacking consistency in the literature across the taxa is the mtDNA starting nucleotide. The human reference genome (NC_012920) base numbering starts in the middle of the control region, whereas murine (NC_005089) and rat (NC_005089) reference genomes start at the edge of the control region, with the first base of the gene for mt-Tf. In the Boore nomenclature [44], mtDNA is opened at MT-CO1. For reasons that appear obvious, the “human” base numbering is impossible to directly extend to species whose mtDNA has more than one control region (e.g., NC_021479) or that lack the control region altogether (e.g., NC_000834). This underscores the need to develop a uniform mitochondrial nomenclature, which would require concerted efforts of investigators from diverse fields.

## 4. Non-Canonical Mitochondrial Genes

In recent years, it became increasingly apparent that in addition to its 37 “formal” genes, mtDNA may encode short open reading frames (ORFs), which can be translated into peptides with important biological functions. The first such peptide, humanin, was identified more than 20 years ago in an unbiased functional screen for clones that protect neuronal cells from death induced by amyloid precursor protein (APP) mutants, which are associated with early-onset familial Alzheimer’s disease [46]. Humanin is encoded by a 75 bp ORF within the gene for MT-RNR2 and was independently isolated in a yeast two-hybrid screen as a partner of the insulin-like growth factor-binding protein-3 (IGFBP-3) [47]. Humanin has since been shown to exert cytoprotective effects against not only mutant APPs but also neuronal cell death induced by other stimuli such as mutant presenilins 1 and 2, and cytotoxic Aβ peptides, Aβ1–42, Aβ1–43, and Aβ25–35 [48]. It has also been shown to protect against IGFBP-3-induced apoptosis [47]. Six other humanin-like peptides were discovered in MT-RNR2. Another short ORF encoding a 16-amino-acid-long mitochondrial open reading frame of the 12S rRNA-c (MOTS-c, reviewed in [49]) has been discovered within the gene for mitochondrial MT-RNR1. This peptide targets skeletal muscle, and its cellular actions inhibit the folate cycle and de novo purine biosynthesis, leading to activation of the AMP-activated protein kinase. MOTS-c treatment in mice prevented age-dependent and high-fat-diet-induced insulin resistance as well as diet-induced obesity [50]. Since then, MOTS-c has been described as an exercise-induced mitochondrial-encoded regulator of age-dependent physical decline and muscle homeostasis [51].

Apart from MT-RNR-encoded small peptides, RNAseq studies established that up to 15% of the mitochondrial transcriptome is made up of long noncoding RNAs (lncRNAs) and micro RNAs, some of which are encoded in mtDNA (micromiRs) [52,53]. At least eight mtDNA-encoded lncRNAs have been identified so far, in addition to small noncoding RNAs and circular RNAs (reviewed in [30,54]). While some of these RNAs have already found their use, e.g., as biomarkers of cardiac remodeling [55], others await the establishment of their bona fides.

## 5. mtDNA Replication

In the mammalian mitochondria, a single DNA polymerase (DNA polymerase γ, POLG) mediates both replication and repair of mtDNA, and consists of a large catalytic subunit and two accessory subunits. The DNA polymerase, 3′ → 5′ exonuclease (proof-reading), and 5′-deoxyribose phosphate (dRP) lyase activities are found in the catalytic subunit. The accessory subunits enhance DNA binding and processivity. The mitochondrial replisome consists of POLG, the mitochondrial single-stranded DNA binding protein (SSBP1), mitochondrial DNA helicase TWINKLE, and includes topoisomerase and RNaseH activities [56]. No dedicated DNA primase activity was described in the mitochondria.

The mode for mtDNA replication remains controversial (reviewed in [57,58]). The original asynchronous strand-displacement model [59–61] suggests that mtDNA replication is primed by an abortive LSP transcript (7S RNA). Interaction of the transcription elongation factor TEFM with POLRMT regulates the balance between priming mtDNA replication and generating a near-genomic-length transcript [62]. Once initiated, replication of the H-strand proceeds unidirectionally over ~70% of mtDNA length until it exposes the origin of the L-strand replication (O_L_). Then, synthesis of a new L-strand is initiated in the opposite direction. This model agrees well with multiple lines of experimental evidence, including the distribution of the de novo point mutations in mtDNA [63].

In the alternative strand-coupled (synchronous) model, there is thought to be a zone of replication initiation within a broad area beyond the D-loop. Within this zone, both strands are synthesized bidirectionally as the conventional double-stranded replication forks advance through continuous synthesis of leading strands and discontinuous synthesis (through Okazaki fragments) of lagging strands. However, this model relies on continuous ligation of Okazaki fragments during the lagging strand synthesis and appears to be inconsistent with recent findings that 100-fold reduction in mitochondrial DNA ligase III does not appreciably affect the rate of mtDNA replication or copy number [64].

The third model is based on the observation of RNA incorporation throughout the lagging strand (RITOLS) [65]. According to this model, replication proceeds as in the strand-displacement model, except the displaced H-strand is present not as single-strand DNA, but rather as DNA/RNA hybrid sensitive to RNAse H, up until it is made duplex by POLG. It is not clear whether RITOLS serve as primers for Okazaki fragments, but it appears unlikely due to the low reliance of mtDNA replication on the quantity of DNA ligase III (see above). Recently, it was demonstrated that the in vivo occupancy profile of mtSSB displays a distinct pattern, with the highest levels of SSBP1 close to the mitochondrial control region and with a gradual decline towards OriL. This pattern correlates with the replication products expected for the strand displacement mode of mtDNA synthesis, thus lending strong in vivo support [66].

## 6. mtDNA Maintenance and Copy Number Control

The copy number of mtDNA molecules per cell varies between tissues; the two extremes of this spectrum are mammalian erythrocytes and sperm, which have no mtDNA and ~5 copies of mtDNA per cell [1], respectively, and oocytes, which may contain >500,000 copies [2]. mtDNA can be eliminated from sperm in the Drosophila male genital tract prior to fertilization, and fertilizing sperm may contain no mtDNA at all, ensuring the uniparental inheritance [67]. In contrast, in mice, uniparental mtDNA inheritance may be facilitated by autophagy of paternal mitochondria in fertilized zygotes [68–70]. Curiously, human oocyte quality directly correlates with mtDNA copy number (mtCN), whereas this correlation is inverse for human sperm [2].

It is important to note that the normal mtCN in a given tissue is not a set figure but can vary over a considerable range. In many studies, mtCN in apparently healthy individuals varies over a 2–10-fold range [71], and mtDNA content between 40–150% of the average is considered clinically normal [72]. The most commonly used techniques for quantifying cellular mtDNA content, qPCR and ddPCR, have resolution limits of approximately 50–60% and 30%, respectively [73]. In samples taken from the same culture over a one-week period, mtCN can vary over the 2–3-fold range [74]. Taking into account this variability, it is unclear how much of the experimentally observed range is attributable to technological difficulties, but it seems obvious that the modest variations in mtCN reported in some studies require thorough validation before being ascribed any biological significance. Apart from the normal variation, mtDNA content can be altered in various pathologic scenarios. mtDNA depletion syndromes [75] are associated with the most dramatic alterations in mtCN, which can drop as much as 50-fold [76]. Such dramatic changes are usually associated with perinatal lethality; however, long survival has been reported in some cases. A 29-year-old patient with 24% residual was observed for this condition since early childhood [77]. In another example, a profound (91%) loss of mtDNA in a 47-year-old patient was associated with relatively mild symptoms such as daytime sleepiness, exercise intolerance, and myalgias in the lower-limb muscles [78]. Therefore, more research is needed to thoroughly delineate the relationship between mtCN and clinical phenotypes.

Contributions of specific proteins to mtCN control remain controversial. The best-studied and most controversial protein in this respect is, perhaps, TFAM. Available evidence suggests that, at least in some experimental systems, mtDNA copy number, mtDNA transcription, and translation of mtDNA-encoded polypeptides, as well as some mitochondrial functions, may closely parallel TFAM expression [79–83]. Therefore, it is often thought that the strictly proportional abundance of TFAM and mtDNA observed in some studies may be dictated by the mutual stabilization of these two components of mitochondrial nucleoids. These views led to a model that describes TFAM’s involvement in mitochondrial biogenesis [84].

Notwithstanding the evidence in support of the close positive correlation between TFAM expression and mtCN, there is a large body of contradictory evidence. TFAM overexpression in flies did not affect mtDNA copy number [85]. In cultured cells, recovery of TFAM levels after ethidium bromide-induced mtDNA depletion lagged behind the recovery of mtDNA copy numbers, suggesting that an increase in mtCN can occur without a proportional increase in TFAM levels [86]. Conversely, a transient TFAM overexpression in cultured cells did not affect the mtDNA copy number [79]. Other investigators observed in developing muscle cells a decrease in mtDNA copy number despite a 4-fold increase in TFAM expression [87 in TFAM levels [86]. Conversely, a transient TFAM overexpression in cultured cells did not affect the mtDNA copy number [79]. Other investigators observed in developing muscle cells a decrease in mtDNA copy number despite a 4-fold increase in TFAM expression [87], indicating that, at least in some settings, increased TFAM expression does not drive increased mtCN. By employing TFAM knockdown and overexpression, we have found that in some cell lines, but not others, mtCN qualitatively, rather than quantitatively, correlates with TFAM expression [88]. Examination of the human protein atlas (www.proteinatlas.org/ENSG00000108064-TFAM/single+cell+type, accessed on 6 June 2022) provides a vivid illustration of the disjunction between TFAM expression and known mtCNs. For example, in the heart, TFAM expression in cardiac myocytes (in which up to 37% of the cellular volume is occupied by mitochondria [89]) is on par or even lower than in endothelial cells, fibroblasts, mixed immune cells, or smooth muscle cells. Finally, and most significantly, in a patient with myoclonic epilepsy with ragged red fibers (MERRF), the tissue with the highest mtDNA copy number had the lowest TFAM levels [90]. Collectively, this evidence indicates that the strong positive relationship between TFAM expression and mtDNA replication observed in some systems is not universal.

Incongruency between TFAM expression and mtDNA copy number deserves attention in the context of the models for mtDNA replication and packaging into nucleoids. Despite the dissenting reports [79,91,92], the prevailing view is that TFAM is present in cells in quantities sufficient to completely cover mtDNA [34,80,81,93,94]. It has also been reported that some cells lacking mtDNA have reduced TFAM expression compared to parental cells containing mtDNA and that the release of TFAM from complexes with mtDNA occurs by Lon-mediated degradation [40]. This appears to suggest a mandatory stoichiometric relationship between TFAM and mtDNA. However, a recent study indicates that in a tissue-specific knockout of the mitochondrial RNA polymerase (PolRmt), TFAM expression remains unchanged despite a severely reduced mtDNA copy number. This TFAM persists free of mtDNA and is not degraded by Lon, which suggests that TFAM/mtDNA stoichiometry is not a universal phenomenon [95]. These observations also suggest that though TFAM may be present in quantities sufficient to completely cover mtDNA, mtDNA in vivo may, in fact, be only partially covered, and a significant pool of “free” TFAM may exist in mitochondria, at least in some scenarios. This latter consideration agrees well with a recent “sliding” model of mtDNA transcription [39] and with the above-cited observations in the MERRF patient [90].

Observations made with some other proteins were also inconclusive. Thus, both increased [96] and decreased [97] skeletal muscle mtDNA content has been reported in patients with mutations in mitofusin 2 (MFN2). While not directly attributable to any particular protein, changes in mtDNA content in the tissues of aged individuals have been widely reported, although the direction of these changes also remains controversial. Some studies report an increased mtDNA copy number in the elderly [98], while others report a decrease and associate frailty with either a lower [99] or higher [100] mtDNA copy number.

In search of the possible mechanisms of mtCN control, sex-specific quantitative trait loci for mtDNA content have been identified on human chromosomes 1, 2, and 3 [101]. Moreover, epigenetic modification of exon 2 of the gene for the catalytic subunit of the mitochondrial DNA polymerase (POLG) has been recently implicated in mtDNA copy number regulation [102]. Despite this progress, cellular mechanisms that govern mtCN control remain largely enigmatic.

In the laboratory setting, mtCN can be reduced by blocking mtDNA replication with intercalating agents such as ethidium bromide and/or POLG inhibitor dideoxycytidine. However, some cells either are naturally resistant to such treatments or may develop resistance during treatment and recover their mtCN [103]. Such treatments are of intrinsically limited utility as they do not allow for the establishment of cell lines with stable mtCN. However, stable cell lines with reduced mtCN can be established by limiting mitochondrial DNA ligase activity by expressing bacterial DNA ligase in the cytosol of cells deficient in DNA ligase III, the only DNA ligase found in mitochondria [104].

## 7. mtDNA Damage, Repair, and Degradation

Compared to the nucleus, the repertoire of DNA repair pathways documented in mitochondria is limited (reviewed in [58,105]). As all polypeptides encoded by mtDNA are components of the OXPHOS system, all mitochondrial functions, including mtDNA repair, depend on proteins encoded in the nucleus, which are translated on cytoplasmic ribosomes and post-translationally imported into mitochondria. Mitochondria are proficient in both short-patch and long-patch subpathways of the Base Excision Repair (BER) pathway. This pathway is responsible for the repair of oxidative and alkylating lesions as well as single-strand breaks in both nuclear (nDNA) and mtDNA [58,105]. Importantly, some evidence suggests that certain oxidized base lesions are repaired more efficiently in mitochondria than in the nucleus [105]. Considering that oxidative mtDNA damage is most frequently mentioned as relevant, it would, perhaps, be inappropriate to state that mitochondria are deficient in DNA repair, at least as far as the repair of the biologically most relevant lesions is concerned. The evidence for the presence of other complete DNA repair pathways in mitochondria remains inconclusive.

**Figure 2.**
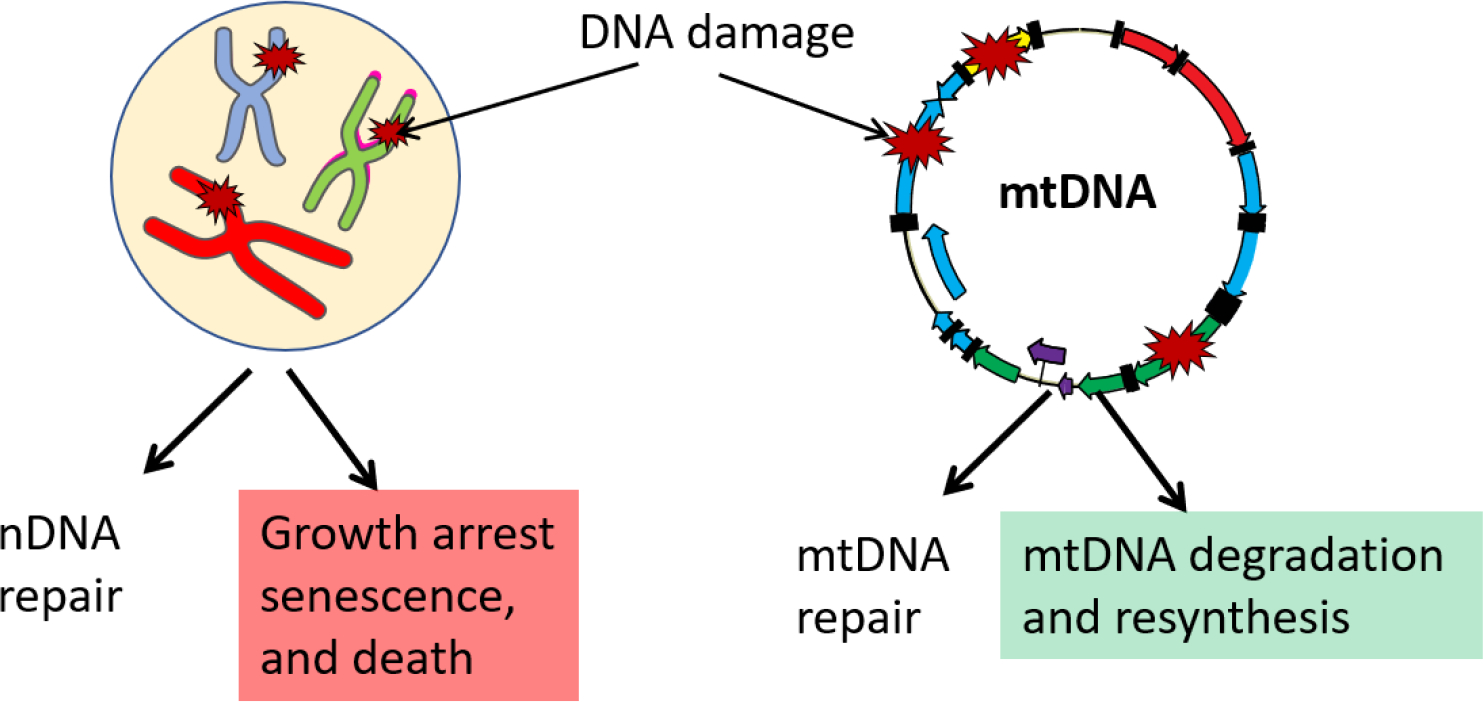
Different cellular and DNA molecule fates upon damage. Inability to repair damage in nuclear DNA (nDNA) is eventually lethal to a normal cell, whereas the multicopy nature of mtDNA enables degradation of mtDNA molecules damaged “beyond repair” and resynthesis of new molecules using intact molecules as templates.

Although mismatch repair (MMR) [106] and double-strand break repair (DSBR) [107] activities have been demonstrated in mammalian mitochondrial lysates, some argued that these results should be interpreted with caution because of the challenges involved in obtaining mitochondrial preparations [108]. How, then, do mitochondria cope with the mutagenic effects of DNA lesions that they are unable to repair? It turns out that in mammalian cells, the high redundancy of mtDNA enables a unique, mitochondria-specific pathway for the preservation of DNA integrity through the degradation of damaged molecules. This pathway is nonspecific to the type of lesion and could be mobilized not only in response to lesions that mitochondria are unable to repair but also in response to the presence of an overwhelming amount of lesions that mitochondria can repair in moderate quantities, such as oxidative lesions [109], abasic sites [110], and gapped duplexes [111] (Figure 2). The kinetics of this process may be different in different cell lines, and in some cell lines, mtDNA loss can be detected as soon as 5–10 min after the challenge with H_2_O_2_ [112]. mtDNA degradation in response to overwhelming damage is well documented and has been used to completely destroy mtDNA in cells and generate so-called ρ^0^ cells [103,110]. For a long time, the enzymatic activities responsible for mtDNA degradation eluded identification. However, recently it has been revealed that mtDNA degradation in cultured human and mouse cells may be mediated by Mitochondrial Genome Maintenance Exonuclease 1 (MGME1) and the proofreading activity of mitochondrial DNA polymerase gamma (POLG) [113,114]. Of note though is that other evidence suggests that in Drosophila spermatogenesis, POLG (Tamas) may mediate mtDNA degradation by mechanisms that do not involve its proofreading activity [115]. While preservation of mtDNA integrity through the degradation of damaged molecules has only been documented in mammalian cells, the ability of Drosophila Tamas to destroy mtDNA during spermatogenesis [115] suggests that similar mechanisms may operate in other taxa.

## 8. mtDNA Transcription: One or Two Heavy Strand Promoters?

At least two promoters are needed to transcribe genes encoded in two mtDNA strands. The existence of a single light strand promoter (LSP) is generally accepted. However, it remains controversial whether there is one or two heavy strand promoters (HSP). Very early on, it has been noted that MT-RNR1 transcript is about 15–60-fold more abundant and is transcribed at a 50–100-fold higher rate compared to the most abundant mRNA transcript encoded by the H-strand [116,117]. Two possible explanations were proposed: (a) the existence of two HSPs, and (b) the premature termination downstream of the mitochondrial 16S rRNA (MT-RNR2). Both models are currently supported by experimental evidence. Two transcription initiation sites were identified in H-strand: one at bp 561 of human mtDNA, 16 nucleotides upstream of the *MT-TF* gene (HSP1), and a second (HSP2) at bp 646, just two nucleotides upstream of the *MT-RNR1* gene inside the *MT-TF* gene (Figure 1) [118–120]. Both promoters are active in an in vitro system; although, in this system the major transcription start site of HSP2 maps to A644 instead C646 [121,122]. An alternative school of thought argues for the existence of a single HSP promoter [123,124]. It has been argued that since mitochondrial transcription termination factor 1 (MTERF1) is dispensable for mouse viability and since in MTERF1 knockout mice no changes in the abundance of putative HSP1 and HSP2 promoter transcripts are observed, MTERF1 cannot selectively stimulate HSP1 transcription by DNA looping as predicted by one of the two HSP-promoter models [120]. However, this argument addresses the mechanism of HSP1 regulation rather than the existence of the HSP2.

Generally speaking, one should exercise caution when extrapolating findings in the human system to those in the murine system and vice versa, even though an assumption that these two systems are regulated in a very similar or identical way appears reasonable. Therefore, it remains possible that there are two HSP promoters in human mtDNA, while in murine mtDNA there is only one. Some evidence suggests that regulation of at least LSP may be different in human and murine cells. Indeed, mLSP has more extensive upstream sequence requirements for maximal transcription in vitro than hLSP does (Figure 1 and [125]). To summarize, available evidence does not conclusively rule out the existence of two separate HSP promoters in human cells in vivo.

## 9. Uniparental mtDNA Inheritance

Animal mtDNA is inherited through the maternal germline with few exceptions (reviewed in [105]). As stated above, in different species, sperm mtDNA can be destroyed either prior to fertilization or after fertilization through autophagy of sperm mitochondria [68–70,115,126]. In either case, the resulting zygote inherits only maternal mtDNA [127]. In most animals, this uniparental mtDNA inheritance is further enforced by a 10,000–100,000-fold dilution of the paternal mtDNA in zygotes (compare, e.g., [1,2]). Despite these formidable mechanisms guarding maternal mtDNA inheritance, accumulating evidence suggests that, in rare cases, paternal mtDNA can be inherited, bringing about another mtDNA controversy [128–134]. Much of the criticism of the rare paternal mtDNA inheritance in humans is centered around the existence of the (hypothetical) mega NUMTs (nuclear sequences resembling mtDNA). It has been argued that these sequences can be artifactually amplified in the course of sequencing library preparation and thus create an impression of heteroplasmy [132,135]. However, Luo et al. reasonably pointed out that their experimental setup was not conducive to such misinterpretation [136]. Our (admittedly superficial) BLAST analysis of the recently released first complete human genome sequence [137], did not reveal any mega-NUMTs that were both complete in length and nearly identical to mtDNA to an extent that would support the notion of possible artifactual nature of the results by Luo et al. [128]. Looking beyond humans, paternal mtDNA inheritance has been described in mice [138] and sheep [139], lending further credibility to the possibility of paternal mtDNA transmission in mammals.

## 10. mtDNA and the Mitochondrial Theory of Aging

Closely related to the subject of mtDNA damage and repair are issues of mtDNA mutagenesis and the role of acquired somatic mtDNA mutations in aging. The progressive accumulation of reactive oxygen species (ROS)-induced somatic mtDNA mutations with age formed the basis for the mitochondrial theory of aging (MTA) in one of several definitions of this theory. Harman first formulated the theory as the free radical theory of aging, in which free radical damage to cellular components was the driving force of aging [140]. Subsequently, it was reported that, in certain conditions in vitro, mitochondria might divert as much as 1–2% of their total electron flow to ROS production [141], which led to the notion of mitochondria being a biologic clock [142]. These high rates of mitochondrial ROS production were obtained with partial oxygen pressure and substrate concentrations much higher than those physiologically obtained, despite mitochondrial rates of ROS production being eventually revised down by an order of magnitude [143–146]. Furthermore, it was recognized that most cellular macromolecules such as proteins, RNA, and lipids are turned over and represent poor candidates for the progressive accumulation of damage with aging. In response to this criticism, Miquel and Fleming [147–149] introduced mtDNA as an oxidative-damage tally keeper. While MTA today is largely abandoned in its form that centers on mtDNA, some of its postulates are still used to justify the ongoing studies. Therefore, it may be useful to review some of these postulates and contradictory experimental evidence that led to their abandonment.

### #1. mtDNA is particularly susceptible to ROS damage because of its close proximity to the electron transport chain, a major cellular source of ROS.

The extent of mtDNA’s accessibility to ROS remains unclear, and without accessibility the proximity appears irrelevant. It has also been demonstrated that mtDNA mutations lack the canonical ROS signature (G>T transversions) [150–152]. This is inconsistent with the leading role of ROS in mtDNA mutagenesis.

### #2. The lack of “protective” histones renders mtDNA more susceptible to mutagenesis.

The protective role of histones has been impossible to elucidate directly in vivo because it is impossible to generate cells knocked out for all histones. Indirect studies indicate that, depending on the experimental system utilized, histones can either sensitize DNA to [153–155] or protect against [156–158] the ROS damage. Importantly, mitochondrial nucleoid proteins can be as protective as histones (reviewed in [105]). Therefore, the notion of histones’ “protective role” is speculative.

### #3. A reduced repertoire of available DNA repair pathways is driving elevated rates of mtDNA mutagenesis.

Base excision repair (BER) pathway is responsible for the repair of the bulk of oxidative DNA damage in both the nucleus and mitochondria. Moreover, oxidative damage in mtDNA may be repaired more efficiently than in the nucleus [159]. mtDNA damage that cannot be repaired is addressed by degradation of the damaged molecule and resynthesis [105,160]. These authors are not aware of any credible experimental evidence proving the notion that mtDNA mutation rates can be reduced by expanding the repertoire of DNA repair pathways available in mitochondria. These observations indicate that the lack of some DNA repair pathways in mitochondria could be inconsequential. Importantly, most substrates for BER and MMR do not induce replicative DNA base mispairing (do not directly lead to point mutations). Rather, they induce mutations due to the low fidelity of DNA polymerases involved in the repair or bypass of these lesions. Therefore, the lack of NER and MMR pathways in mitochondria may play a protective role against mutations, as counterintuitive as it may sound. Indeed, in the absence of NER, damaged mtDNA molecules are presumed to be channeled for degradation [161], thus avoiding error-prone repair. Therefore, the notion that a reduced repertoire of available DNA repair pathways is driving elevated rates of mtDNA mutagenesis is speculative.

### #4. The “vicious cycle” of DNA damage and ROS production drives mtDNA mutagenesis.

The “vicious cycle” hypothesis is based on the assumption that the majority of mtDNA mutations result in mitochondrial dysfunction. This dysfunction, in turn, is necessarily accompanied by increased ROS production. This arrangement results in a “vicious” feed-forward cycle. This concept is incompatible with the experimentally observed unresponsiveness of ROS production to elevated levels of mtDNA mutations in mito-mice [162–164].

## 11. Extramitochondrial mtDNA

Zhong et al. described circulating cell-free mtDNA (mtDNAcf) in blood plasma more than two decades ago. [165]. This extramitochondrial mtDNA species has been suggested to have prognostic value in cancer, cardiac arrest, and severe sepsis [166,167]. Subsequently, mtDNAcf was identified as a major mediator of innate immunity and systemic inflammatory response syndrome (SIRS). In response to tissue damage (e.g., blunt-force trauma) mtDNA is released into plasma by an unknown mechanism. This results in toll-like receptor 9 (TLR9)-mediated neutrophil activation and systemic inflammation [168]. Controversially, while TLR9 is believed to be exclusively activated by DNA that lacks CpG methylation, a number of studies reported mtDNA methylation and even identified DNMT1 as a putative mediator of this methylation [169–175]. It is possible, however, that severe hypomethylation of mtDNA mediates its specific recognition by TLR9 [176,177].

Unexpectedly, mtDNA was also reported in the cytosol. Both strong insults (e.g., oxidative stress, bacterial or viral infection, etc.) and altered compaction of mtDNA in nucleoids resulting from TFAM haploinsufficiency were shown to promote cytosolic mtDNA release. [178]. This intracellular release of mtDNA has been implicated in cell-intrinsic innate immune responses [179,180]. Mechanistically, the cytosolic release of mtDNA could be mediated by Bax/Bac-mediated herniation of the inner mitochondrial membrane [181–183].

## 12. Conclusions

Our understanding of mtDNA and its contribution to biological processes continues its exponential growth. Things that were unthinkable less than two decades ago, such as mtDNA DAMPS, mtDNA control of the innate immunity, and mtDNA-derived peptides, are now an everyday reality. Yet, many basic mechanistic puzzles related to mtDNA replication, copy number control, transcription, etc., have proved remarkably difficult to solve and have bred controversy. Many of those difficulties are secondary to the insufficient resolution power of currently available analytical techniques. However, the continuously growing repertoire of new analytical and genetic technologies available to investigators bears the promise of resolving current controversies and even greater discoveries in the near future.
